# Morphogens and growth factor signalling in the myeloma bone-lining niche

**DOI:** 10.1007/s00018-021-03767-0

**Published:** 2021-02-11

**Authors:** Emma V. Morris, Claire M. Edwards

**Affiliations:** 1 Nuffield Department of Surgical Sciences, University of Oxford, Oxford, UK; 2 Nuffield Department of Orthopaedics, Rheumatology and Musculoskeletal Sciences, Botnar Research Centre, University of Oxford, Old Road, Oxford, OX3 7LD UK

**Keywords:** Multiple myeloma, Bone, Osteoblast, Endosteal niche, Growth factors

## Abstract

Multiple myeloma is a malignancy caused by the clonal expansion of abnormal plasma cells. Myeloma cells have proven to be incredibly successful at manipulating their microenvironment to promote growth and to evade modern therapies. They have evolved to utilise the integral signalling pathways of the bone and bone marrow to drive disease progression. The bone marrow is often described in the context of a single structure that fills the bone cavity and supports normal haematopoiesis. However, within that structure exists two anatomically different niches, the perivascular niche and the endosteal niche. These contain different cell types functioning to support normal immune and blood cell production as well as healthy bone. These cells secrete numerous signalling molecules that can influence myeloma cell biology and behaviour. The endosteal niche is home to specific bone cell lineages and plays a pivotal role in myeloma cell establishment and survival. This review will concentrate on some of the signalling pathways that are hijacked by myeloma cells to shape a favourable environment, and the different influences myeloma cells are exposed to depending on their spatial location within the bone marrow.

## Introduction

Multiple myeloma (MM) is an incurable malignancy caused by the clonal expansion of abnormal terminally differentiated plasma cells in the bone marrow. Once established MM cells have a remarkable ability to manipulate their environment causing a significant uncoupling of bone remodelling [1]. Bone homeostasis is normally governed by an ongoing and dynamic process of bone resorption followed by new bone formation. MM cells disrupt this process, promoting increased bone resorption and suppressing bone formation, thereby resulting in the formation of osteolytic bone disease. These lesions often cause debilitating complications, such as bone pain, pathological fractures, spinal cord compression, hypercalcemia and generalised osteoporosis. Currently, it is standard practice to treat MM patients with bisphosphates or similar anti-resorptive therapies to attempt to minimise further bone loss. However, even though these therapies protect against further damage, there is presently no treatment to restore pre-existing bone loss. Furthermore, since there is no successful treatment that can totally eradicate MM cells from the bone marrow, patients eventually relapse leading to further bone destruction [2].

MM is a genetically complex and heterogenous disease resulting from multiple genetic events that drive tumour development and progression. Most MM cases either harbour translocations involving the immunoglobulin heavy chain (IgH) locus, which results in the expression of juxtaposed genes including Cyclin D1 (CCND1, 11q13), FGF3/MMSET (4p16.3), Cyclin D3 (CCND3, 6p21), MAFC (16q23), and MAFB (20q11) [3], or are hyperdiploid, characterised by multiple chromosomal gains, preferentially trisomy of chromosomes 3, 5, 7, 9, 11, 15, 19, and 21 [1, 4]. These tumour initiating events are thought to occur to drive cell cycle entry by deregulating D-type cyclins, which is a crucial pathogenic event in the malignant transformation of plasma cells [5]. In addition, several secondary genetic and epigenetic abnormalities have been identified as drivers of disease progression. These include changes in DNA methylation, mutations in the MAPK pathway, and P53, as well as genetic and epigenetic abnormalities in the components of the nuclear factor-kappa B (NF-κB) and wingless-type (Wnt) pathway [4, 6]. Despite this complex level of heterogeneity, one element that almost all MM tumours have in common is their dependence on the bone marrow microenvironment, or bone marrow niches [7]. It is becoming increasingly recognised that the different niches within the bone play an important role in the pathogenesis and chemoresistance of the disease. The different niches support and facilitate the cell survival and expansion of these mutant cells allowing them to thrive. The spatial localisation of myeloma cells within the marrow influences their behaviour, this is in part due to the types and concentrations of molecules the MM cells are exposed to, which are secreted by their neighbouring cells. Furthermore, there is evidence to suggest that MM cells that reside close to the bone surface (endosteal niche) may be afforded protection from chemotherapy, ultimately leading to relapse [8].

## Bone marrow niches

The bone marrow microenvironment is not only a scaffold that provides structure, it is also a critical support network for haematopoiesis, made up of multiple cell types including haematopoietic stem and progenitor cells, mesenchymal stem cells, osteoblasts, osteoclasts, stromal cells, endothelial cells, adipocytes and immune cells; these cells influence each other to create a dynamic and balanced environment whereby the end product is normal healthy immune cell production and haematopoiesis. Within that environment exists at least two anatomically different niches. The central or perivascular niche, which is in the inner part of the bone marrow, and the bone-lining or endosteal niche, which is in close proximity to the bone surface. The central niche is the larger of the two comprising  > 90% of the bone marrow volume and harbours 85% of the haematopoietic stem cells (HSCs) [9]. It is highly vascular with a high density of endothelial cells, multipotent mesenchymal stromal cells (MSCs), and smooth muscle cells [10]. The endosteal niche is much smaller located at the interface between the bone and the bone marrow it is enriched with the remaining 15% of HSCs, as well as cells of specific bone cell lineages, such as osteoblast, osteoclasts, and osteocytes. The type of blood vessels present in the two niches also differs with the sinusoids and arterioles running through the central niche and the transition zone vessels which join the sinusoid and arterioles together being part of the endosteal niche [8]. The central niche also houses a larger proportion of immune cells and is the more active, nutrient-rich part of the bone marrow being responsible for daily blood production, therefore just by geography alone the influences that are exerted on infiltrating MM cells by neighbouring cells are different dependent on where they reside. The central niche is also sensitive to radiation therapy whereas the endosteal niche is mainly preserved following ablation and seems important for subsequent haematopoietic regeneration [11]. It would appear that the endosteal niche is more protected from incoming insults and can act as a place of refuge for MM cells arriving in the bone. They can settle in the niche in a mitotically dormant state, allowing single tumour cells time to adapt to a new environment whilst avoiding the effects of drugs that target proliferating cells. In time, these cells become active again, taking advantage of the factors that are secreted by resident cells such as interleukin (IL-6), insulin-like growth factor (IGF), hepatocyte growth factor (HGF), vascular endothelial growth factor (VEGF), stromal cell-derived factor 1α (SDF1α) and a proliferation-induced ligand (APRIL), which provide crucial signals for tumour growth and survival [7, 12]. Furthermore, MM cells are highly decorated with the cell surface marker heparan sulfate proteoglycan (HSPG) syndecan-1 (CD138) which facilitates communication with the cells of the niche by providing docking sites for bioactive molecules, such as secreted cytokines, chemokines and growth factors, promoting both signal transduction and adhesion [13, 14]. Moreover, MM cells secrete macrophage inflammatory protein (MIP)-1α and β which in combination with receptor activator of nuclear factor-kappaB ligand (RANKL) and IL-6 enhance osteoclastogenesis [15]. Thus, MM cells are able to shape the microenvironment to favour tumour growth resulting in niche remodelling and the development of osteolytic bone disease [16]. In normal adult homeostasis, the endosteal surfaces are lined with quiescent ‘bone-lining cells’ which become activated to differentiate into matrix-secreting osteoblasts in response to required bone remodelling [17–19]. However, in the presence of active MM, this process is disrupted causing impaired osteoblast differentiation and function resulting in an inability to form new bone. This coupled with the increase in osteoclast number and function [20] tips the finely balanced bone remodelling process towards bone destruction and the formation of osteolytic bone lesions. The mechanisms by which MM cells change the microenvironment are orchestrated through interactions with many different cell types as well as the dysregulation of numerous signalling pathways. A number of these pathways have a spatial element to them, these spatial interactions may explain why MM cells that reside in the endosteal niche are more robust against therapeutic strategies.

## Morphogenic pathways

Several morphogenic pathways have been implicated in osteoblast suppression, osteoclast activation and MM cell survival, such as the Wnt pathway, TGFβ/BMP pathways, and Notch signalling. Morphogens are molecules that are secreted locally in a specific region establishing a concentration gradient. This gradient determines the development of cells which respond differently to different concentrations of the morphogen, this mechanism of signalling is critical for normal embryonic development. As morphogens move through tissues, they bind receptors displayed on cell surfaces and initiate intracellular signalling cascades that result in the activation of transcriptional effectors. Different levels of effector activate and repress different sets of genes [21]. Cells positioned within the morphogen gradient react to concentration differences by modification of their differentiation program inducing cell-fate specification. These molecules highlight the importance of spatial interaction within the tissue, these same spatial interactions can also be key drivers of disease. MM cells that reside in the endosteal niche close to osteoblasts and osteoclasts can take advantage of the secretion of these morphogen molecules from these resident cells whereby cells in the central niche may be exposed to lower or even negligible concentrations, these differences may give MM clones which have arisen from or established in the endosteal niche a survival advantage. It is the combination of bidirectional signalling within this niche between MM cells and bone marrow stroma and bone cells that drives disease.

## Wnt signalling in the endosteal niche

The Wnt signalling pathway is a highly conserved signal transduction pathway that plays a crucial part in embryonic development as well as in a variety of cellular processes, such as the regulation of proliferation, cell-fate determination, migration, and cell polarity. There are 19 Wnt ligands which can bind and activate their cell surface-associated Frizzled (FZD) receptors. Wnt signals can be transduced via either the well-defined ‘canonical’ Wnt/β-catenin pathway, or by either of two ‘non-canonical’ β-catenin-independent signalling cascades, termed the Wnt/PCP and Wnt/Ca^2+^ signalling pathway [22, 23]. Wnt ligands are cysteine-rich glycoproteins which are secreted by osteoblasts and are relatively unstable leading to constraints in long-range signalling. Therefore, they typically act as niche or stem cell factors [24]. The activation of canonical Wnt signalling is followed by β-catenin translocation into the nucleus where, associated with Tcf/Lef transcription factors it triggers the expression of target genes [25]. The Wnt signalling cascade plays a pivotal role in osteoblastogenesis and bone formation, it increases bone mass through a number of mechanisms including renewal of stem cells [26], stimulation of pre-osteoblast replication, induction of osteoblastogenesis [27] and inhibition of osteoblast and osteocyte apoptosis [28]. Wnt signalling is tightly regulated by several secreted antagonists, such as soluble Frizzled-related proteins (sFRPs), these interfere with Wnt/Frizzled receptor binding, and Dickkopf (DKK) proteins and sclerostin which bind to their co-receptors LDL-related protein (LRP)5/6 [25]. MM cells secrete all three Wnt inhibitors giving them an arsenal of molecules by which to suppress osteoblast function. DKK1 is known to inhibit the canonical pathway by binding to LRP5. sFRP-2, has been found to be constitutively produced by MM cells of patients with advanced bone lesions, and the removal of sFRP-2 from MM cell-conditioned media restored osteoblast differentiation and maturation supporting sFRP-2’s role in impaired bone formation [29, 30]. sFRP-3 has also been implicated in MM as a Wnt signalling inhibitor found to be upregulated in some MM cells [30]. Although sclerostin is predominantly produced by osteocytes embedded in newly formed bone, it is also found to be produced by MM cells and has been found to be highly expressed in patients with MM, with circulating concentrations correlating with disease stage and fractures [31]. Sclerostin is a potent inhibitor of osteoblast differentiation and mineralisation of pre-osteoblastic cells [32] exerting its inhibitory effects by binding to LRP5 as well as LRP6 [33]. The disruption of the Wnt pathway is central to the osteoblast suppression observed in MM. As Wnt ligands exert their effects locally, the exposure to these ligands and the inhibitory effects that MM cells bestow on this signalling pathway may cause differences in MM cell biology dependent on their spatial relationships to their neighbouring cells. Another signalling pathway that is known to have extensive crosstalk between Wnt signalling is the bone anabolic bone morphogenetic protein pathway.

## Bone morphogenetic proteins

Bone morphogenetic proteins **(**BMPs) are potent growth factors, they constitute the largest subgroup of the transforming growth factor (TGF-β) family of ligands, there are thought to be around 30 different BMPs, signalling is complex with numerous receptors and ligands and is highly dependent on cell type and context. BMPs exert their signalling through a heteromeric receptor complex, composed of type I and type II receptors at the cell surface that transduces intracellular signalling through the phosphorylation of the SMAD 1/5/8 complex, which translocates to the nucleus and either alone or in combination with other non-SMAD transcription factors regulates the transcription of target genes. As their name suggests, they play a pivotal role in orchestrating morphogenesis and patterning throughout the body. Moreover, due to their osteogenic effects, studies have shown that systemic administration of recombinant BMP-2, BMP-6, or BMP-7 improves bone mass [34–36]. However, they also play a role in a number of other processes, such as cellular lineage commitment, differentiation, proliferation, cellular maintenance and survival [37, 38]. Furthermore, in MM, BMP signalling has been proposed to influence processes, such as growth control, bone homeostasis, iron metabolism and angiogenesis [39]. In general, MM cells express all the BMP receptors except for ALK1, suggesting that stimulation by BMP ligand binding may convey a survival advantage. However, studies have found that BMP-2, 4, 5, 6, 7 and 9 are toxic to MM cells causing cell cycle arrest and apoptosis. Moreover, high levels of BMP-6 in primary MM cells predicted superior overall survival in patients undergoing high-dose chemotherapy [40]. Since BMPs are also known mediators of bone formation, they appeared to have potential not only to suppress MM growth and survival but also to encourage new bone formation, thereby restoring bone in these patients. However, many of these toxic observations were made in vitro using MM cells in single cultures [41–44]. Though, in in vivo, there is evidence to suggest that MM cells can overcome BMP-induced toxicity. A study by Gooding and colleagues looked at the effect of BMP inhibition in vivo using transcriptomic profiling of the myeloma-bone-lining niche, they revealed that the interaction of tumour cells with stromal cells abrogated BMP-induced MM cell death and that BMP blockade reduced osteoblast suppression thereby improving bone disease in a murine model [45]. These observed in vivo effects highlight the importance of the bone microenvironment. MM cells appear to evade BMP-induced apoptosis by utilising the protection of neighbouring cells. BMP signalling can be blocked by a number of different antagonists, such as gremlin, noggin, follistatin and sclerostin, all of which are secreted by osteoblasts; therefore, the local concentration of BMPs and extracellular antagonists is important for the ultimate cellular response [46]. Another BMP inhibitor is sclerostin domain containing 1 (Sostdc1) which inhibits BMP-2 and -7 and is expressed at low levels in MM and osteoblast lineage cells when cultured separately. However, when these cells are cultured together in co-culture, its expression is significantly induced in both cell types [47]. BMP-2, -4 and -7 have been shown to be highly expressed in the endosteal niche compared with the central marrow [48]. Therefore, MM cells in the endosteal niche may elicit a protective relationship from neighbouring osteoblasts coupled with the likely exposure to higher concentrations of locally secreted antagonists. BMPs may play a role in slowing MM cell growth; however, it is the combination of complex signalling and direct cellular contacts that governs the fate of these infiltrating cells. BMPs also stimulate osteoclast differentiation and activity [49]. It may be that in the context of MM bone disease, the positive osteogenic effects of BMP signalling are so far diminished by the dysregulation of osteoblast function and decrease in number, that it has a negative impact, whereby BMPs further stimulate osteoclast differentiation and activity promoting further bone destruction.

## Notch signalling pathway

Another important signalling pathway that is dysregulated in several malignancies including MM is the Notch pathway. The Notch family includes four transmembrane receptors (Notch1-4), which are activated by ligands, Jagged (Jagged1, 2) and Delta-like (Dll1, 3, 4) [50]. Notch signalling is known for its crucial role in cell-fate decision, tissue patterning and morphogenesis [51]. It is important in skeletal development and remodelling whereby it drives osteoclast differentiation [52]. Notch receptors are expressed on multiple cell types including endothelial cells [53], osteocytes [54], osteoblasts [55], bone marrow stromal cells and osteoclasts as well as MM cells [52]. Furthermore, in the context of MM, Notch signalling has been shown to be important in cell localisation into the bone marrow, proliferation, survival, and drug resistance. Circulating MM cells are recruited to the bone marrow by a chemotactic gradient involving the SDF-1/CXCR4 axis [56]. Upon arrival in the bone marrow, MM cells activate Notch signalling via the overexpression of Jagged1/2 ligands, inducing resident osteocytes and stromal cells to increase the production of osteogenic factors, such as the receptor activator of NF-κB ligand (RANKL). This causes a change in the ratio between RANKL and its decoy receptor osteoprotegerin, thereby resulting in an increase in osteoclast formation. MM cell-mediated activation of Notch also increases sclerostin production by osteocytes resulting in a decrease in Wnt signalling and inhibition of osteoblast differentiation, thereby exacerbating bone destruction. Notch signalling has been shown to be bidirectional, the reciprocal activation of Notch signalling in MM cells has been shown to enhance MM proliferation and survival. Moreover, the increase in bone resorption caused by the uncoupling of bone remodelling releases growth factors from the bone matrix, such as TGF-β, which also contribute to MM cell long-term survival and proliferation. Again, the impact upon each MM cell may change depending on their spatial localisation as Notch signalling is preferentially activated by direct cell–cell contact. Furthermore, paracrine Notch activation appears to be mediated by different receptors depending on the cell type interaction, stromal cells activated Notch 1 and 2 whereas osteocytes activate Notch 3 and 4, suggesting that regulation of Notch activation in MM is mediated by complex paracrine interactions with different cell types within the bone/bone marrow microenvironment [54]. Notch signalling has also been implicated in the development of drug resistance in MM. Jagged ligand overexpression in MM cells triggers hyperactivation of Notch signalling in both MM cells and the surrounding bone marrow stromal cells (BMSCs), promoting MM cell expression of pro-survival factors, such as BCL2 and Survivin [57]. Aberrant Notch signalling is by no means confined to the endosteal niche, MM activated Notch signalling also plays a fundamental role in the central marrow influencing endothelial cell behaviour. Overexpression of Jagged 2 was shown to correlate with the downregulation of miR-223 in BMSCs resulting in the upregulation of both IL-6 and VEGF leading to increased MM proliferation and angiogenesis [58]. MM cells activate several proangiogenic pathways in endothelial cells through the release of growth factors, such as VEGF and HGF, cell-to-cell contacts prompt endothelial cell migration, chemotaxis, adhesion and spreading, leading to the formation of an enhanced angiogenic network able to cope with the increased oxygen demand of the expanding tumour [59].

## Exosomes

Ligand-receptor and direct cell-to-cell interactions are generally thought of as the predominant means of intercellular communication. However, in the last few decades, the release of specific extracellular vesicles from the cell has been identified as an important mediator of intercellular communication that plays a regulatory role in cancer establishment and progression. There has been particular interest in the participation of exosome signalling between tumour cells and the microenvironment. Exosomes are a subpopulation of extracellular vesicles that mediate intercellular communication both locally and systemically. They are actively secreted by a wide range of cell types and contain cell-specific bioactive molecules, such as proteins, lipids and nucleic acids. They exert their functions by transferring their cargo to the target cells either by endocytosis or direct fusion with the cell membrane [60]. This horizontal transfer of molecular or/and genetic information ensures continuous communication among both neighbouring and distant cells. In the context of MM, this ensures constant crosstalk between MM cells and cells of the microenvironment, supporting MM pathogenesis by promoting immunosuppressive effects, angiogenesis, osteolysis and drug resistance [61]. The main producers of these exosomes that contribute to MM pathogenesis are BMSCs as well as MM cells themselves. These cell types produce large quantities of exosomes that affect a variety of target cells reprogramming the recipient cell’s function to create a pro-tumour environment that can support disease progression. Target cells include natural killer cells, myeloid-derived suppressor cells, mesenchymal cells, endothelial cells, osteoblasts and osteoclasts [62]. miRNAs are among the most important signalling molecules which are sorted into exosomes, and the miRNA profile found in MM patients can differ quite considerably compared to healthy individuals. MM patients exhibit elevated levels of miRNAs, such as miR-21 [63], miR-146a [64], let-7b and miR-18a [65], which promote MM growth and survival, while others, such as the tumour suppressor miR-15a, are markedly reduced [66]. Hypoxic conditions are also shown to increase exosome production. The bone marrow is hypoxic in nature, however, as MM cells proliferate the levels increase. With chronic hypoxia, exosomes secreted by MM cells enhance angiogenesis by targeting factor-inhibiting hypoxia-inducible factor-1 (HIF-1) via miR-135b [67]. Moreover, cells under stress are reported to increase their exosome production impacting on several different pathways, BMSC-derived exosomes influence the activation of several survival pathways, such as c-Jun N-terminal kinase, p38, p53, and Akt, as well as promote the migration of MM cells through the up-regulation of CXCL12/CXCR4/monocyte chemoattractant protein-1 (MCP-1) axis [68]. Exosomes are produced under normal physiologic conditions by all live cells; thus, this is an important communication pathway throughout the bone microenvironment spanning both the endosteal and perivascular niches. As with many modes of cellular communication, MM cells utilise this selective delivery mechanism in an autocrine, paracrine and endocrine manner to further shape their surroundings promoting a tumour-supportive environment.

## Dormancy

One area of increasing interest in the biology and evolution of MM is cellular dormancy. This process is the result of extrinsic signalling from the tumour microenvironment which leads to a state of quiescence, characterised by minimal proliferation, minimal cell death and reversibility [69]. In normal bone homeostasis, it is thought that one of the roles of the endosteal niche is to induce and fuel HSC dormancy. The ability of MM cells to thrive is particularly dependent on ‘hijacking’ normal processes within the bone microenvironment, the utilisation of the induction of a dormant stem-like state is no exception. MM cells arriving in the bone seed on the bone surface in the endosteal niche and interact with bone-lining cells, this induces a mitotically dormant state akin to HSC dormancy whereby single tumour cells can pause and adjust to new and sometimes hostile environmental factors, such as hypoxia, changes in extracellular matrix signalling or endoplasmic reticulum stress. Dormant cells can remain co-existing with resident cells of the bone microenvironment for years. However, over time as osteoclasts remodel the bone-lining niche, MM cells are dislodged from the bone surface and become reactivated [16]. Thus, dormancy is a huge challenge for the use of conventional therapies, such as anticancer drugs and radiation, which target actively proliferating cells. Dormant cells may persist as minimal residual disease years after treatment has ended which in time results in disease relapse. Using murine models, dormant MM cells have been shown to express a distinct transcriptome signature characterised by the expression of immune genes largely associated with myeloid cell lineage differentiation. By targeting one of the most highly expressed genes, the tyrosine kinase AXL, which binds to the receptor GAS6 expressed by osteoblasts, dormant MM cells were reactivated and tumour burden was increased in vivo [70]. Once dormancy has been interrupted, these cells can then be targeted using traditional methods. Prostate cancer cells that disseminate to the bone exhibit the same dormant behaviour when in contact with osteoblasts. They too express AXL and it is thought that it is the AXL-GAS6 axis is in part responsible for their entry into dormancy [71]. More work is needed to further understand the mechanisms surrounding the induction of tumour cell dormancy. Imaging techniques, such as two-photon microscopy and intravital imaging, have been employed to study and track dormant tumour cells in murine models [16, 48, 70]. However, dormancy has been difficult to study in humans, new techniques are emerging that may begin to increase our knowledge of spatial interactions making it possible to start to study this rare population of cells within the human bone marrow.

## Techniques to study MM cells in the bone marrow

Studying the structure and cellular composition of the bone/bone marrow can be challenging. Many studies have used murine models to study the evolution of MM cell biology and disease progression. MRI, micro-CT or ultrasound can image bone structure; however, these techniques are limited by their low resolution at the cellular level. Higher-resolution images can be achieved using fluorescently labelled cells which can be tracked by fluorescent microscopy giving a snapshot of spatial interactions at the single-cell level [16]. Both mouse and human samples have been used for flow and mass cytometry for single-cell proteomics, identifying changes in cell populations [72], as well as transcriptomic profiling by single-cell RNA sequencing, to shed light on transcriptional changes that occur in the bone marrow niches that support MM growth and survival [45, 73]. These are powerful techniques that provide a wealth of information; however, one limitation they have in common is that they use cell suspensions, therefore any spatial information is lost. New imaging techniques using next-generation imaging, such as the fluorescence-based microscopy systems CODEX® (CO-Detection by indEXing) and Vectra® Polaris® Automated Quantitative Pathology Imaging System or the mass cytometry system, Hyperion, are starting to bridge this gap using single-cell technology combined with high-multiplex imaging on intact tissue [74]. These advanced techniques can label a large number of proteins in parallel on a single section while preserving cellular morphology and tissue architecture. Using this approach, it will be possible to map and decode the precise cellular and spatial relationship between MM cells and the cells of the BM microenvironment. New in vitro models have also been developed to mimic both niche environments. A 3D bone marrow model containing two sub-niches was created using 3D bioprinting technology. A bioprintable calcium phosphate cement scaffold with seeded osteogenic multipotent mesenchymal stromal cells was used to model the endosteal niche, and Matrigel containing both endothelial progenitor cells and MSCs to model the perivascular niche. Primary CD138+ myeloma cells were then introduced to the models, cultured and analysed for both survival and proliferation. Interestingly, the 3D bone marrow model with combined sub-niches significantly increased the proliferation of CD138+ myeloma cells compared to both environments separately [75]. This supports the notion that both niches are essential in supporting myeloma cells. These new techniques will facilitate the discovery of novel and important cellular relationships within these specialised niches and reveal new therapeutic targets, while increasing our knowledge of spatial interactions in the progression of myeloma.

## The relationship between the niches

The research that has been carried out studying the different components of the bone marrow niches has clearly demonstrated that the extrinsic signalling that MM cells are exposed to depending on their spatial positioning has a profound effect on MM cell biology and clonal expansion. Both the endosteal and central niches in the marrow can support the proliferation of MM cells. However, the endosteal niche is the primary niche that supports dormant stem-like functions of MM cells. Residing near the bone surface provides a level of drug resistance, not due to location alone, more likely it is governed by cell cycle status which is controlled by extrinsic signalling from neighbouring cells. It is thought that MM cells preferentially reside in the endosteal niche, at least when they first arrive in marrow [48]. For many years, it was believed that a gradient of decreasing oxygen levels exists from the perivascular niche to the endosteal niche [76], playing a fundamental role in maintaining this dormant stem-like state. However, evidence obtained by in vivo measurements of local oxygen tension (pO_2_) in the BM of live mice seemed to indicate that the lowest pO_2_ (1.3%) was found in deep peri-sinusoidal regions, while the endosteal region is less hypoxic (1.8%) [77]. More studies are needed to fully understand changes in oxygen available in different zones of the bone and how bone marrow hypoxia and MM cells affect each other. However, what is clear is that increased hypoxia within the growing tumour stimulates a program of enhanced angiogenesis [78]. Once MM cells have been displaced from the endosteal surface and move into the perivascular niche they come into contact with different cells types, such as adipocytes, which release adipokines into the environment which fuel MM cell migration, proliferation and survival [79], as well as endothelium cells and mural cells which create a microenvironment rich in angiogenic factors and cytokines that promote MM growth and spread. [10]. Evidence suggests that MM cells promote angiogenic activity via HIF-1α, leading to the overproduction of cytokines, such as VEGF, angiopoietin-1 [80], and osteopontin [81]. VEGF stimulates proliferation and chemotaxis in stromal cells via VEGF receptor-1, and in endothelial cells via VEGFR-2. VEGFR-1 also induces MM cell proliferation and facilitates VEGF secretion enhancing angiogenesis and contributing to MM cell growth and survival by inducing IL-6 secretion [82]. As with many cell types, and illustrated by VEGF signalling MM cells have a bidirectional relationship with endothelial cells, transcriptomic profiling showed that 251 genes were preferentially expressed in endothelial cells from myeloma-bearing mice compared with controls [45]. Furthermore, tumour cells support abnormal vessel structure and growth through both angiogenic and non-angiogenic mechanisms. Thereby both niches appear to play a role in sustaining and promoting disease progression [10].

## Conclusion

**Fig. 1:**
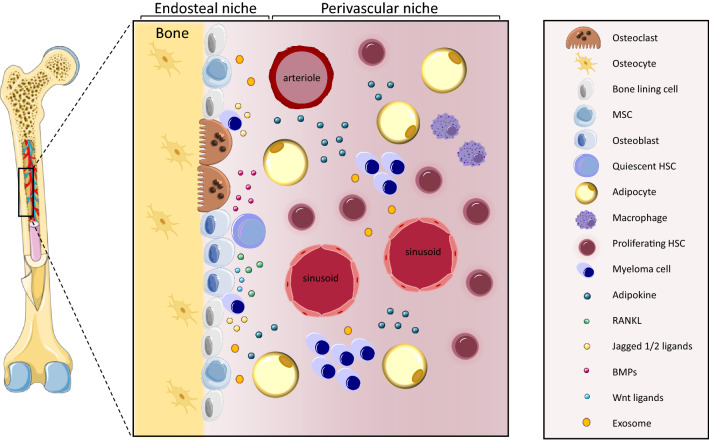
Endosteal and perivascular niches. Myeloma cells thrive in the bone marrow by utilising important communication pathways within their microenvironment via direct interactions, soluble factors, and exosome release. Myeloma cells settle on the endosteal surface where long-term multipotent cells reside, cells, such as HSCs, MSCs and bone-lining cells alongside osteoblasts and osteoclasts, where they maintain a dormant like state. Once MM cells become displaced from the bone surface, they move into the nutrient-rich perivascular niches where they are exposed to different cell types, such as adipocytes and endothelial cells, which release growth factors promoting proliferation and expansion. Some of the elements in this illustration were adapted from Servier Medical Art, provided by Les Laboratoires Servier (available at: https://smart.servier.com)

The complexity of the signalling pathways and cell–cell interactions between MM cells and their neighbouring cells within the bone microenvironment is vast (Fig. 1). This makes targeting the MM cells alone an ineffective strategy. Future work which focuses on identifying important spatial relationships and interaction in the bone marrow may uncover new novel targets that may interrupt the parasitic symbiotic relationship that MM cells have with their environment. Increasing our knowledge of how MM cells are able to shape the endosteal landscape could lead to new combination therapies that target the microenvironment and the signalling pathways within it to make the bone marrow a more hostile less permissive environment for these cells to thrive in.
